# Modified Rosi–Cahill technique after left extended colectomy for splenic flexure advanced tumors

**DOI:** 10.1007/s10151-024-02956-w

**Published:** 2024-07-20

**Authors:** J. J. Segura-Sampedro, J. Cañete-Gómez, A. Craus-Miguel

**Affiliations:** 1 General and Digestive Surgery Service, La Paz University Hospital, 28046 Madrid, Spain; 2 School of Medicine, University of the Balearic Islands, Health Research Institute of the Balearic Islands, Palma de Mallorca, Spain; 3 General and Digestive Surgery Service, Valme University Hospital. School of Medicine, University of Seville, Seville, Spain; 4 General and Digestive Surgery Service, Son Espases University Hospital, Palma, Spain

**Keywords:** Cytoreductive surgery, Colectomy, Oncologic surgery, Oncologic colectomy, Quality of life

## Abstract

Advanced splenic flexure tumors are uncommon and have a higher risk of relapse. To ensure that the resection includes the entire area of lymphatic drainage with a complete mesocolic excision (CME), a left extended colectomy is needed. In peritoneal carcinomatosis, there is often extensive involvement of the sigma and splenic flexure of the colon. In many instances, total colectomies are chosen for these patients, even when a significant portion of the colon could be preserved. The potential impact on quality of life after splenic flexure colon resection is discussed, as well as the importance of anatomical knowledge and expertise in performing this type of surgery. Overall, this work presents a modified technique that aims to improve the outcomes and quality of life for patients with splenic flexure colon cancer. Creating a tension-free anastomosis after extended left-sided colorectal resection is challenging. There is a negative impact on quality of life when an ileorectal anastomosis is created. The colorectal anastomosis performed after modified Rosi–Cahill or Deloyers’ technique allows reduced small bowel bacterial overgrowth, achieves better water and sodium absorption, and altogether permits improved stool consistency. There are potential advantages of the Rosi–Cahill technique over other popular options such as Deloyers’ procedure as there is no torsion of the ileocolic vessels and no mesenteric windows. A video was recorded showing a potential pitfall during Deloyers’ technique resulting in the creation of a mesenteric window. The proper rotation of the colon during the modified Rosi–Cahill procedure was also filmed. Overall, this work presents a modified technique for reconstruction after left extended colectomy that aims to improve the outcomes and quality of life for patients with splenic flexure colon cancer.

**Supplementary Information:**

The online version contains supplementary material available at 10.1007/s10151-024-02956-w.

## Introduction

Splenic flexure colon cancer is rare making up only 2–5% of all colon cancers. It has a high likelihood of causing obstruction [1]. A recent international study revealed that splenic flexure colon cancer is more likely to cause stenosis, infiltrate the serosa, have a mucinous histology, and relapse as peritoneal carcinomatosis [2]. In peritoneal carcinomatosis, there is often extensive involvement of the sigma and splenic flexure of the colon. In many instances, surgeons opt for total colectomies for these patients, even when a significant portion of the colon could be preserved [3–5].

Creation of a tension-free anastomosis after an extended left-sided colorectal resection can be challenging. In order to avoid tension between the remaining bowel ends many surgeons elect to perform a subtotal or total colectomy. However, this results in sacrifice of the ileocecal valve and subsequent deterioration of bowel function with frequent bowel movements which persist in the long term and have an adverse effect on quality of life [6, 7].

Another popular option is Deloyers’ procedures which produces a torsion of ileocolic vessels. We describe the anticlockwise right colon to rectum anastomosis (Rosi–Cahill technique [8]) and discuss advantages and differences with Deloyers’ technique.

## Oncologic extended left colectomy with middle colic and inferior mesenteric lymphadenectomy

With minor variants the blood vessels that supply the splenic flexure are the left colic artery (LCA) and the left branch of the middle colic artery (MCA). However, the mesentery surrounding the splenic flexure is referred to as an avascular region, and blood flow from the marginal artery, known as Griffiths’ point, can be inadequate in some cases [1]. This low blood supply might be responsible for the higher rate of anastomotic leak in segmental colectomies.

Hohenberger et al. [9] introduced the concept of complete mesocolic excision (CME) in radical colic resection. Like total mesorectal excision in patients with rectal cancer, CME aims to remove the entire mesocolon in one piece. Studies have shown that CME results in more thorough lymph node removal, fewer cases of cancer recurrence, and improved long-term outcomes compared to non-CME surgery [10]. In a recent meta-analysis, segmental resection for transverse colon tumors (non-CME), including those in the splenic flexure, was associated with lower lymph node yields, higher anastomotic leak rates [6], and worse stool consistency [11, 12].

On the basis of histopathological results, prospective observational studies, and theoretical concepts, surgeons performing advanced colon cancer resections should adhere to the following principles of radicality:

- Lymph node metastases travel along the vascular supply, primarily with the paracolic supply, up to 10 cm from the macroscopic edge of the primary tumor. Thus at least 10 cm of the colon should be removed if vascular division is radicular [13].
- The extent of resection is determined by the vascular supply and the consequently defined area of lymphatic drainage. In principle, if the tumor is located between two major vessels, both should be divided centrally [13].
- In the special case of advanced splenic flexure tumors, with elevated risk of node metastasis, both pedicles, middle colic vessels, and inferior mesenteric vein and artery define the area of lymphatic drainage. As complete mesocolic excision in patients with colon cancer has improved overall survival and progression-free survival in some cohort studies, both pedicles should be included in the resection when sufficient expertise and training in colon surgery make it possible, especially in those tumors which are likely to have lymph node metastasis [9].

## Reconstruction after extended left colectomy: Deloyers’ technique

**Fig. 1:**
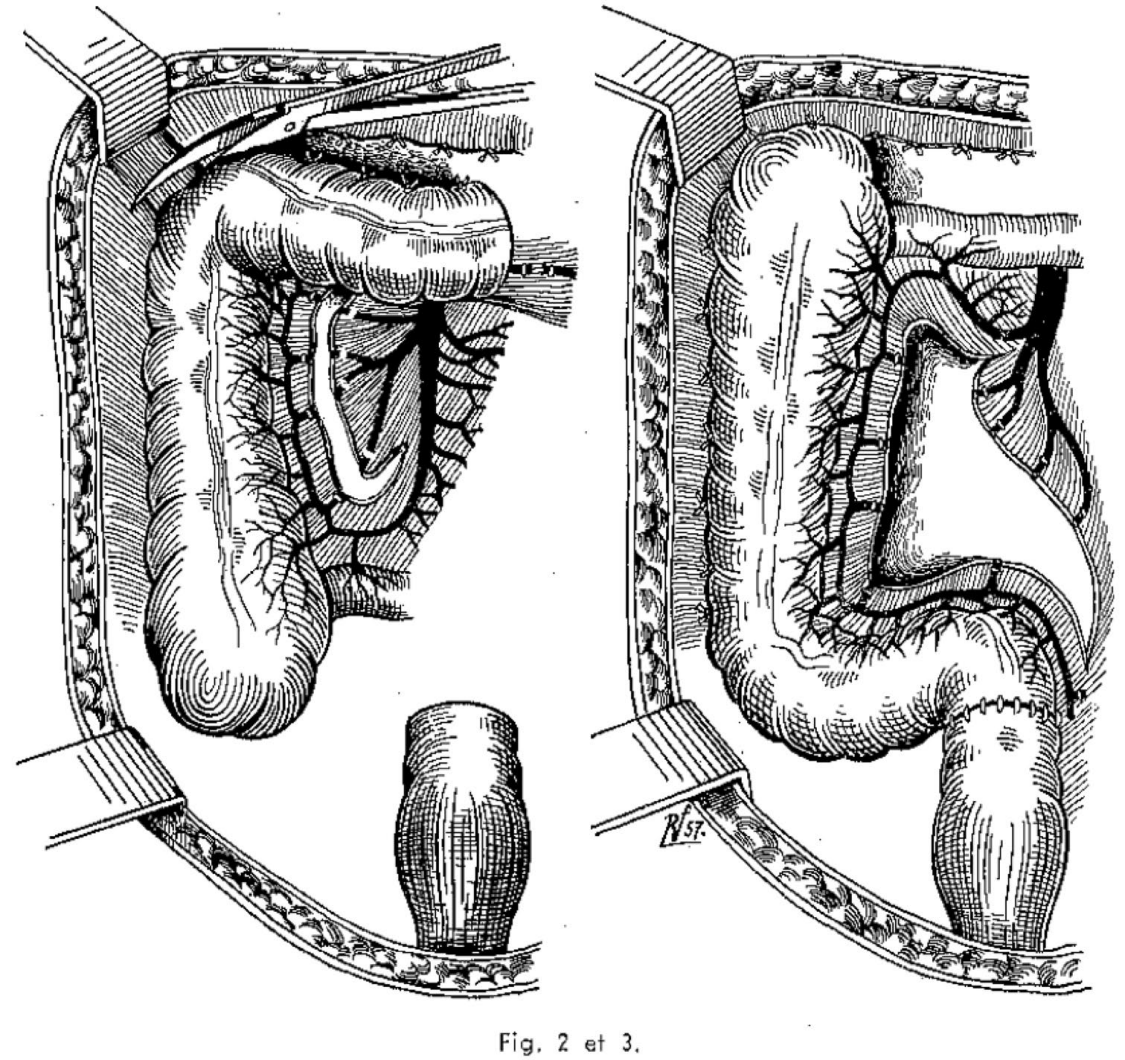
Original depiction of Deloyers’ technique in 1964 [14]

Deloyers’ procedure was described in 1964 [14] and has been popularized in recent years with satisfactory results. This technique allows an anastomosis between the right colon and the rectum. After complete mobilization of the right colon, it is then rotated 180° counterclockwise about the axis of the ileocolic vessels for a colorectal or coloanal anastomosis (Fig. 1) [12, 15].

**Fig. 2:**
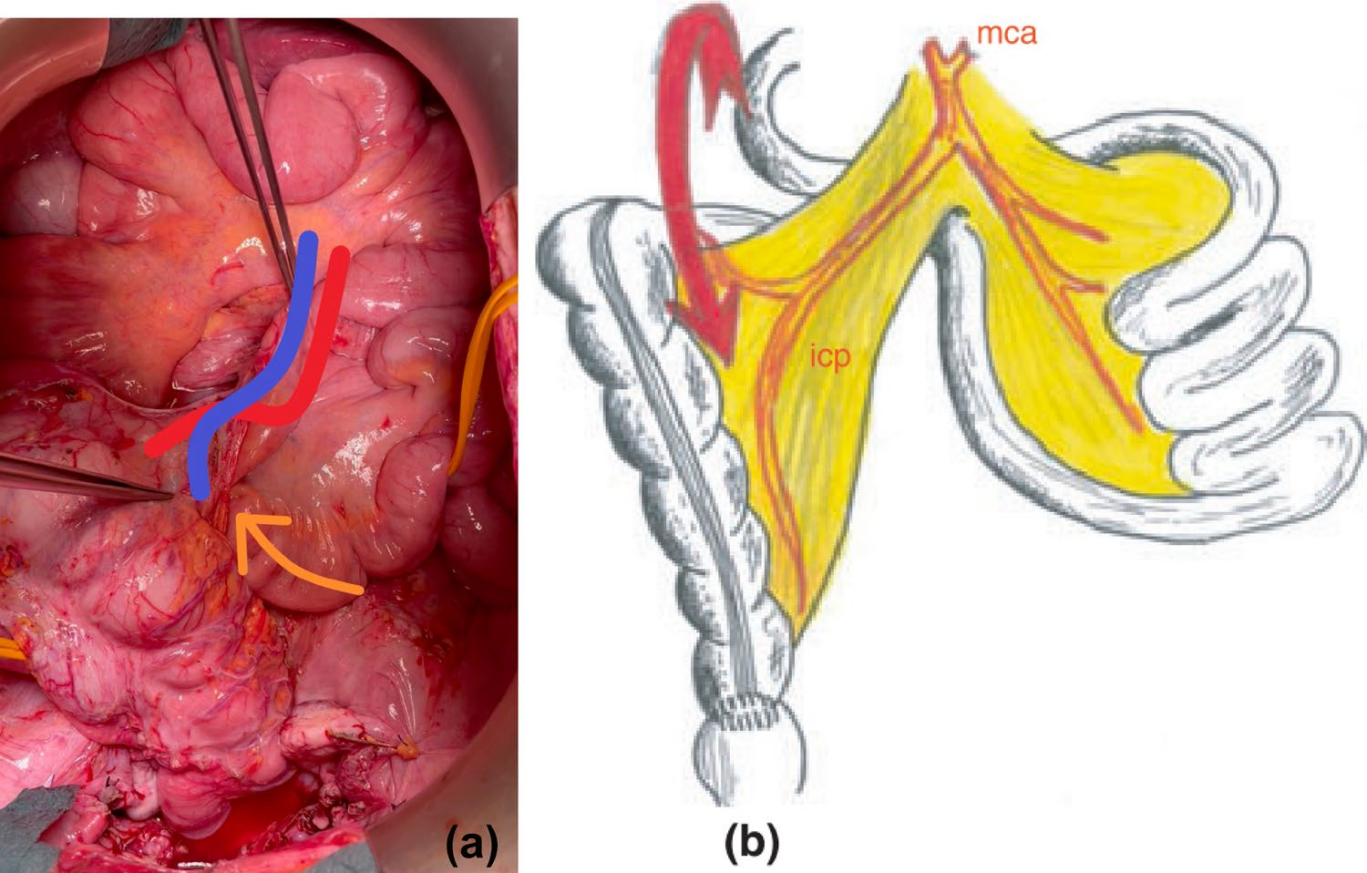
**a** Pitfall in Deloyers technique when rotation is performed clockwise: a mesenteric window is created. **b** Depiction of this technique taken from Dumont et al. [18]

This technique produces a 180° torsion of the ileocolic axis. A counterclockwise rotation around the ileocolic vessels is performed [16] to avoid a mesenteric window. A pitfall of this technique develops when rotation is performed clockwise: a mesenteric window is created through which the terminal ileum might slide, thereby creating an internal hernia (Fig. 2) (Video 1) [17, 18]. This technique has been popularized as it requires less dissection, and it is easier to perform laparoscopically. Although good results have been reported we do not recommend it because of the aforementioned problems.

There are other technical options which involve transmesenteric approach [19, 20] with even higher risk of internal hernia and torsion that we would not recommend either, as a there is a technique available without any vessel torsion nor mesenteric windows.

## Reconstruction after extended left colectomy: Rosi–Cahill technique

**Fig. 3:**
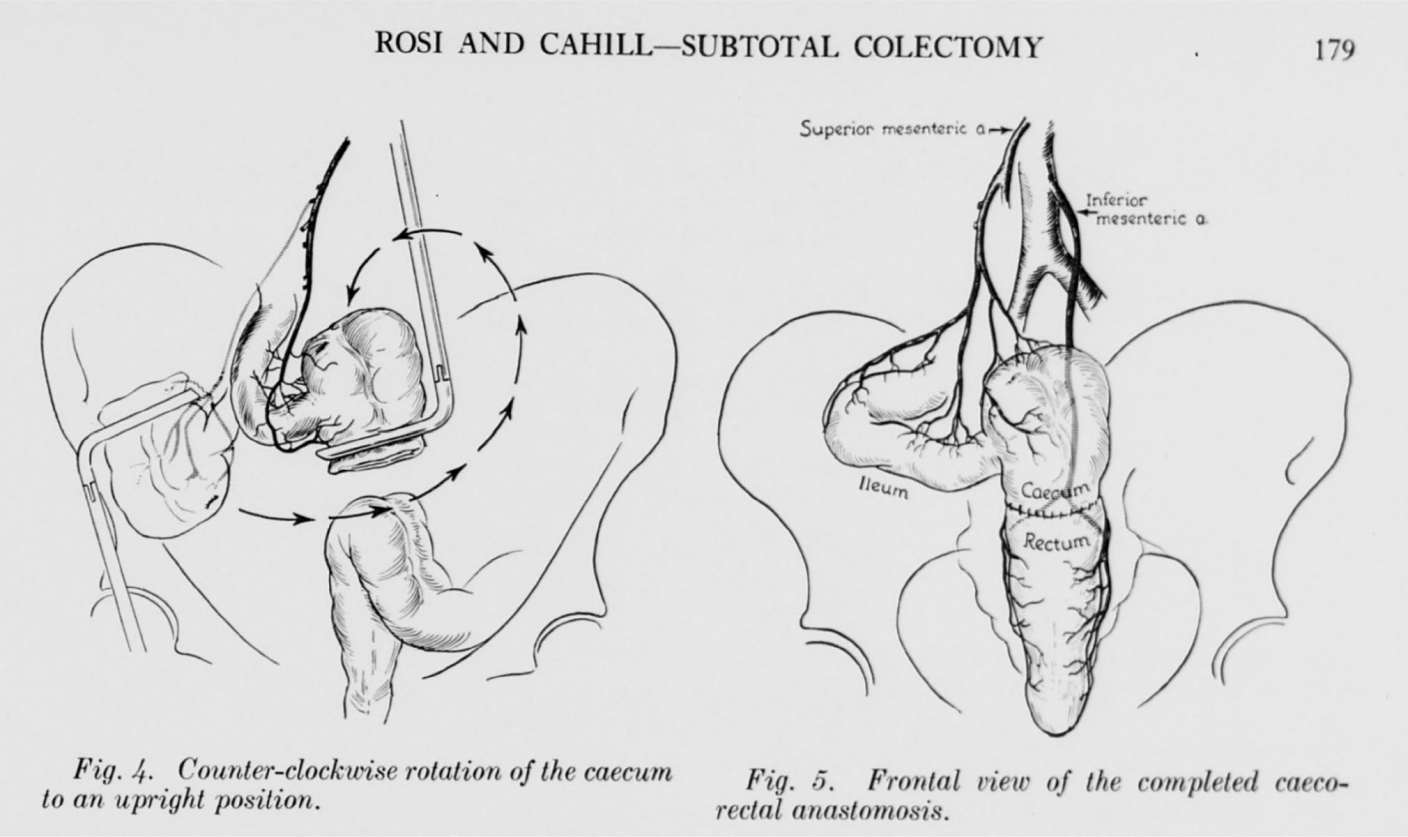
Original depiction of Rosi–Cahill subtotal colectomy in 1960 [8]

**Fig. 4:**
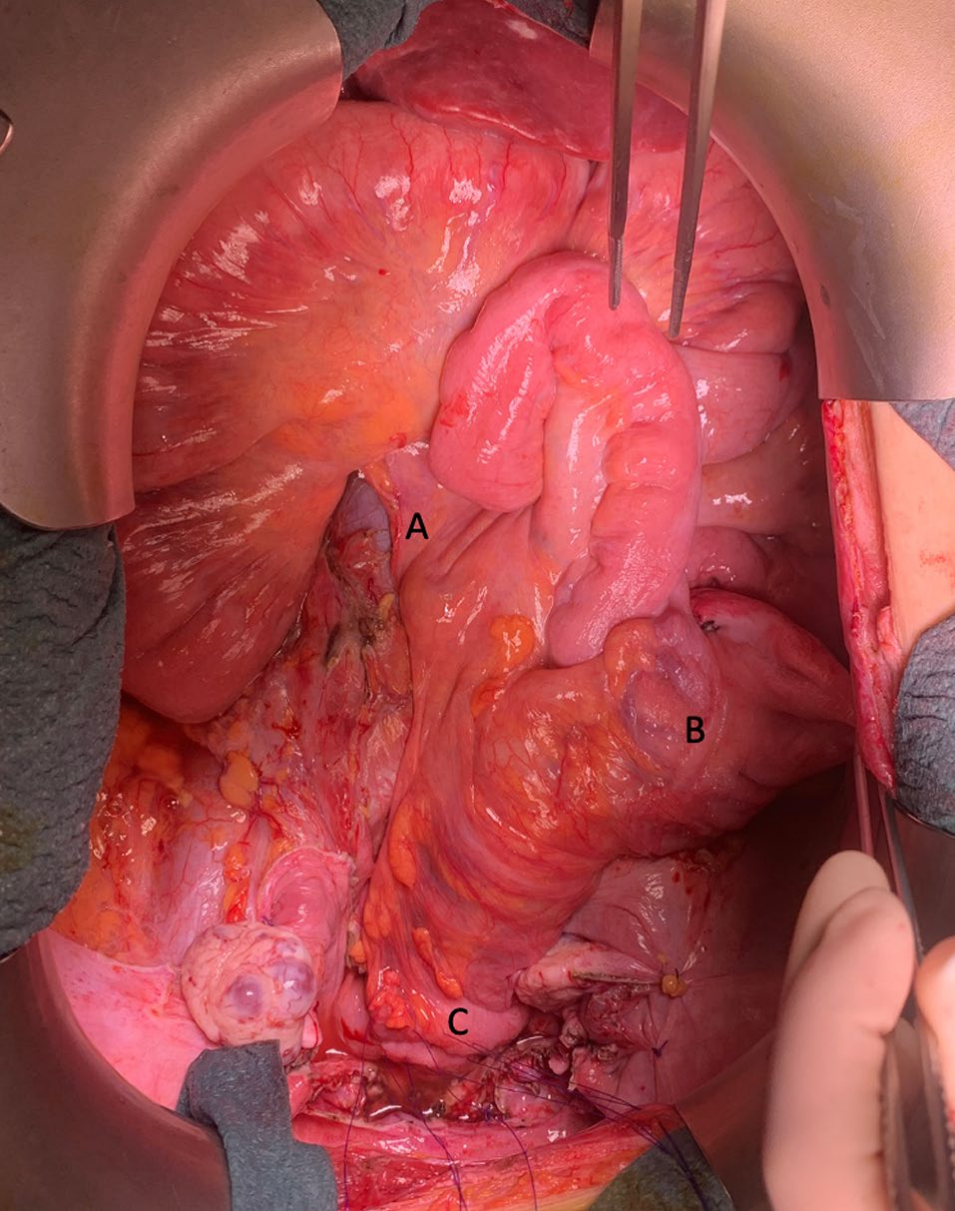
Modified Rosi–Cahill technique to achieve tension-free colorectal anastomosis after extended left colectomy without torsion of the mesenteric vessels or mesenteric windows. A nontorsioned ileocolic vessels, B cecum in left iliac fossa, C tension-free colorectal anastomosis

Rosi and Cahill described this technique in 1960 [8] as a cecorectal anastomosis, 4 years before Deloyers’, for subtotal cecum-preserving colectomies (Figs. 4 and 5 in [8]; see Fig. 3). The modified Rosi–Cahill technique preserves the right colon and requires more dissection than Deloyers’ technique but has the advantage of not leaving any mesenteric window and the avoidance of mesenteric vessel rotation. It has been wrongly named Deloyers [21] or wrongly described in several studies [17]. This makes the current literature on these techniques questionable.

This procedure is started by a right medial visceral rotation, using a Cattell–Braasch maneuver. A complete mobilization of the right colon, the hepatic flexure, and the entire mesenteric root is achieved. Retroperitoneal organs, including the inferior vena cava (IVC), the right renal pedicle, the right iliac vessels, the duodenum, and the head of the pancreas are exposed.

The greater omentum is detached from the transverse colon at the hepatic flexure. The right colic artery and the middle colic artery are divided at their origins. Inferior mesenteric vein is transected under the pancreas and inferior mesenteric artery at its origin. The colon is divided from the rectum with no preservation of superior rectal artery. As a result of the complexity of a future appendectomy, the appendix is systematically removed [15].

A 31-mm anvil of a circular stapler is introduced in the ascending colon. An anticlockwise rotation of the right colon is then performed, and a colorectal end-to-end stapled anastomosis is created (Fig. 4) (Video 2). As a result of this rotation, the cecum changes its location to the mesogastric region, and all the small bowels remain in the right flank as if the embryologic decussation had never happened.

## Improved quality of life and nutrition

Creation of a tension-free anastomosis after an extended left colectomy can be challenging and requires expertise and anatomical knowledge [6]. In order to avoid tension between the remaining bowel ends many surgeons elect to perform a subtotal or total colectomy. However, this results in deterioration of bowel function with frequent bowel movements which persist in the long term and have an adverse effect on quality of life [22].

Moreover, in cases of peritoneal carcinomatosis, multiple intestinal resections (15–45%) are often added to the colonic resection (35–60%) [23, 24], thereby increasing the risk of short bowel syndrome [25]—a significant concern. Cytoreductive surgery, whether accompanied by hyperthermic intraperitoneal chemotherapy (HIPEC) or not, comes with a notable risk of morbidity. It is widely recognized that the presence of poor nutritional status plays a significant role in contributing to this morbidity. Specifically, malnutrition emerges as a major factor leading to readmissions in these situations [26]. Hence, preserving the colon not only promises an improved quality of life but also contributes to better nutritional status, reduced sarcopenia [27], and, presumably, enhanced tolerance to oncological treatment, thereby impacting overall survival [28].

There are several procedures that allow an anastomosis between right colon and rectum that avoids subtotal or total colectomy. These techniques preserve the ileocecal valve, thereby conferring better functional outcomes compared with ileorectal anastomosis (IRA) as it is associated with variable rates of morbidity, bowel dysfunction, and decreased quality of life [29, 30]. Conservation of the terminal ileum and the ileocecal valve has also physiological advantage of avoiding small bowel bacterial [31] and fungal overgrowth [32], better water and sodium absorption, altogether permitting improved stool consistency [22, 33].

## Conclusion

Both splenic flexure colon cancer and its involvement in peritoneal carcinomatosis present challenging scenarios. Left extended colectomy, combined with the modified Rosi–Cahill technique, shows potential for improving both oncological and quality of life outcomes, especially in advanced cases, with a diminished likelihood of adverse effects. This approach seems to be a viable and safer alternative compared to Deloyers’ technique, allowing for a complete rotation of the right colon and a tension-free colorectal anastomosis while minimizing the risk of torsion of ileocolic vessels and mesenteric windows.

## Supplementary Information

Below is the link to the electronic supplementary material.

Supplementary file1 (MP4 33439 KB)

Supplementary file2 (MP4 89193 KB)

## Data Availability

Data sharing not applicable to this article as no datasets were generated or analyzed during the current study.
